# Supplementary material for: TIAM-1 differentially regulates dendritic and axonal microtubule organization in patterning neuronal development through its multiple domains
Source: PLoS Genet. 2022 Oct 12;18(10):e1010454. doi: 10.1371/journal.pgen.1010454 (PMC9612824; doi:10.1371/journal.pgen.1010454)
Supplement: S1 Table — (DOCX) [file pgen.1010454.s007.docx]

**S1 Table. List of strains, plasmids, and oligonucleotides used in this study**

**Strains used in this study:**

| Strain | Genotype |
| --- | --- |
| TV12922 | *wyEx5216[Pdes-2::gfp::rab-3; Pser2prom3::myr::mCherry;* *Podr-1::rfp]* [1] |
| STR369 | *hrtSi41[Pdes-2::unc-33L::gfp; Punc-119::unc-119] I ;unc-119(ed3) III* [2] |
| BRC0566 | *unc-119(ed9) III; antIs31[attp-phiC31-f; Cbr-unc-119(ant40); Pglh-2::phiC31; rol-6(partial); Pmyo2::GFP; attp-phiC31-r] II*  BRC0566 is a gift from John Wang lab [3]. *ant40* is a 691 bp deletion allele by CRISPR/Cas9 in the original *Cb-unc-119* marker gene on *antIs31*. |
| OCY295 | *ntuIs1[Pdes-2::tomm-20::gfp; Pdes-2::myr::mCherry ; Podr-1::gfp]X* [1] |
| OCY580 | *tiam-1(ok772) I; ntuIs1[Pdes-2::tomm-20::gfp; Pdes-2::myr::mCherry ; Podr-1::gfp] X* |
| OCY690 | *mig-2(mu28) X; ntuIs1[Pdes-2::tomm-20::gfp; Pdes-2::myr::mCherry ; Podr-1::gfp] X* |
| OCY854 | *tiam-1(ok772) I; wyEx5216[Pdes-2::gfp::rab-3; Pser2prom3::myr::mCherry;* *Podr-1::rfp]* |
| OCY875 | *tiam-1(ok772) I; ntuIs1[Pdes-2::tomm-20::gfp; Pdes-2::myr::mCherry ; Podr-1::gfp] X; ntuEx31[Pdes-2::TIAM-1B; Pmyo-2::gfp]* |
| OCY1014 | *ntuEx138[Pdes-2::unc-104::gfp; Pdes-2::myr::mCherry; Podr-1::gfp]* |
| OCY1094 | *tiam-1(ok772) I; ntuIs1[Pdes-2::tomm-20::gfp; Pdes-2::myr::mCherry ; Podr-1::gfp] X; ntuEx276[Pdes-2::TIAM-1C; Pmyo-2::gfp]* |
| OCY1141 | *tiam-1(ok772) I; ntuIs1[Pdes-2::tomm-20::gfp; Pdes-2::myr::mCherry ; Podr-1::gfp] X; ntuEx275[Pdes-2::TIAM-1A; Pmyo-2::gfp]* |
| OCY1223 | *pfn-1(ok808) I; ntuIs1[Pdes-2::tomm-20::gfp; Pdes-2::myr::mCherry ; Podr-1::gfp] X* |
| OCY1224 | *unc-60(su158) V; ntuIs1[Pdes-2::tomm-20::gfp; Pdes-2::myr::mCherry ; Podr-1::gfp] X* |
| OCY1313 | *ntuSi1 [Pdes-2::TIAM-1A::gfp; Punc-119::unc-119] II in* BRC0566 |
| OCY1320 | *tiam-1(ok772) I; ntuIs1[Pdes-2::tomm-20::gfp; Pdes-2::myr::mCherry ; Podr-1::gfp] X; ntuEx277[Pdes-2::TIAM-1N539; Pmyo-2::gfp]* |
| OCY1322 | *tiam-1(ok772) I; ntuEx138[Pdes-2::unc-104::gfp; Pdes-2::myr::mCherry; Podr-1::gfp]* |
| OCY1353 | *fhod-1(tm2363) I; ntuIs1[Pdes-2::tomm-20::gfp; Pdes-2::myr::mCherry ; Podr-1::gfp] X* |
| OCY1400 | *wve-1(ok3308) I; ntuIs1[Pdes-2::tomm-20::gfp; Pdes-2::myr::mCherry ; Podr-1::gfp] X* |
| OCY1503 | *ntuEx305 [Pdes-2::gfp; Pser2prom3::tagRFP::tba-1; Podr-1::gfp]* |
| OCY1504 | *ntuEx306 [Pdes-2::gfp; Pser2prom3::tagRFP::UtrCH; Podr-1::gfp]* |
| OCY1538 | *ntuSi1 [Pdes-2:: TIAM-1A::gfp; Punc-119::unc-119] II; ntuEx282[Pdes-2::tag::tba-1; Podr-1::gfp]* in BRC0566 |
| OCY1608 | *tiam-1(ntu14) I; ntuIs1[Pdes-2::tomm-20::gfp; Pdes-2::myr::mCherry ; Podr-1::gfp] X* |
| OCY1629 | *ntuSi9 [Pdes-2::unc-119::mCherry; Punc-119::unc-119] II* in BRC0566 |
| OCY1630 | *ntuSi1 [Pdes-2:: TIAM-1A::gfp; Punc-119::unc-119] II; ntuEx279[Pdes-2::unc-119::mCherry; Podr-1::gfp]* in BRC0566 |
| OCY1675 | *ntuIs1[Pdes-2::tomm-20::gfp; Pdes-2::myr::mCherry ; Podr-1::gfp] X; ntuEx281[Pdes-2::unc-119; Pmyo-2::gfp]* |
| OCY1949 | *tiam-1(ntu22) I; ntuIs1[Pdes-2::tomm-20::gfp; Pdes-2::myr::mCherry ; Podr-1::gfp] X* |
| OCY1987 | *tiam-1(ok772), hrtSi41[Pdes-2::unc-33L::gfp; Punc-119::unc-119] I; unc-119(ed3) III* |
| OCY2034 | *ntuEx263[Pdes-2::gfp::UtrCH; Pser2prom3::tagRFP::tba-1; Podr-1::gfp]* |
| OCY2041 | *act-3(st15) V; ntuIs1[Pdes-2::tomm-20::gfp; Pdes-2::myr::mCherry ; Podr-1::gfp] X* |
| OCY2042 | *tiam-1(ok772) I; ntuIs2[Pdes-2::unc-116::gfp; Pdes-2::myr::mCherry; Pmyo-2::gfp] Ⅲ* |
| OCY2103 | *wsp-1(gm324) VI; ntuIs1[Pdes-2::tomm-20::gfp; Pdes-2::myr::mCherry ; Podr-1::gfp] X* |
| OCY2109 | *hrtSi5[Pdes-2::ebp-2::gfp] I* [4] |
| OCY2128 | *ntuIs2[Pdes-2::unc-116::gfp; Pdes-2::myr::mCherry; Pmyo-2::gfp] Ⅲ* [1] |
| OCY2129 | *ntuIs13[Pdes-2::myr::mCherry; Podr-1::gfp] I* |
| OCY2130 | *ntuIs13[Pdes-2::myr::mCherry; Podr-1::gfp] I; ntuEx278[Pdes-2:: TIAM-1A::gfp ; Pmyo-2::gfp]* |
| OCY2131 | *ntuSi9 [Pdes-2::unc-119::mCherry; Punc-119::unc-119] II ; ntuEx278[Pdes-2::TIAM-1A::gfp ; Pmyo-2::gfp]* in BRC0566 |
| OCY2132 | *ntuSi1 [Pdes-2::TIAM-1A::gfp; Punc-119::unc-119] II; ntuEx283[Pdes-2::tagRFP::UtrCH; Podr-1::gfp]* in BRC0566 |
| OCY2133 | *ntuSi9 [Pdes-2::unc-119::mCherry; Punc-119::unc-119] II; ntuEx284[Pdes-2::TIAM-1A::gfp; Podr-1::gfp]* in BRC0566 |
| OCY2134 | *ntuIs13[Pdes-2::myr::mCherry; Podr-1::gfp] I ; ntuEx285[Pdes-2::VN173; Pdes-2::VC155; Pmyo-2::gfp]* |
| OCY2135 | *ntuIs13[Pdes-2::myr::mCherry; Podr-1::gfp] I ; ntuEx286[Pdes-2::unc-119::VN173; Pdes-2::TIAM-1A::VC155; Pmyo-2::gfp ]* |
| OCY2136 | *unc-119(ed3) III; ntuIs1[Pdes-2::tomm-20::gfp; Pdes-2::myr::mCherry ; Podr-1::gfp] X* |
| OCY2137 | *tiam-1(ok772) I; unc-119(ed3) III; ntuIs1[Pdes-2::tomm-20::gfp; Pdes-2::myr::mCherry ; Podr-1::gfp] X* |
| OCY2138 | *tiam-1(ok772) I; ntuSi9 [Pdes-2::unc-119::mCherry; Punc-119::unc-119] II* in BRC0566 |
| OCY2181 | *tiam-1(ok772), hrtSi5[Pdes-2::ebp-2::gfp] I* |
| OCY2202 | *tiam-1(ok772), ntuIs13[Pdes-2::myr::mCherry; Podr-1::gfp] I ; ntuSi14 [Pdes-2::TIAM-1A::gfp; Punc-119::unc-119]* in BRC0566 |
| OCY2231 | *tiam-1(ok772), ntuIs13[Pdes-2::myr::mCherry; Podr-1::gfp] I* |
| OCY2232 | *tiam-1(ok772), ntuIs13[Pdes-2::myr::mCherry; Podr-1::gfp] I ; ntuSi15 [Pdes-2::TIAM-1B::gfp; Punc-119::unc-119] II* in BRC0566 |
| OCY2233 | *tiam-1(ok772), ntuIs13[Pdes-2::myr::mCherry; Podr-1::gfp] I ; ntuSi16 [Pdes-2::TIAM-1C::gfp; Punc-119::unc-119] II* in BRC0566 |
| OCY2234 | *tiam-1(ok772), ntuIs13[Pdes-2::myr::mCherry; Podr-1::gfp] I ; ntuSi17 [Pdes-2::TIAM-1N539::gfp; Punc-119::unc-119] II* in BRC0566 |
| OCY2235 | *ntuSi1 [Pdes-2::TIAM-1A::gfp; Punc-119::unc-119] II; act-4 (dz222) X ; ntuEx283[Pdes-2::tagRFP::UtrCH; Podr-1::gfp] II* in BRC0566  *act-4 (dz222)* is a kind gift from Hannes E Bülow lab. |

***** *Podr-1::gfp* and *Pmyo-2::gfp* were used as co-injection markers.

**List of mutant alleles used in this study**

| Mutant allele | Description |
| --- | --- |
| *act-3(st15), mig-2(mu28), unc-60(su158), wve-1(ok3308), fhod-1(tm2363), wsp-1(gm324), pfn-1(ok808), tiam-1(ok772), tiam-1(ntu14), tiam-1(ntu22), act-4(dz222), and unc-119(ed3)* | We identified the *act-3(st15)* allele as a missense mutation in ACT-3(Y144N), which was reported as a gain-of-function allele and caused the enrichment of actin filaments in muscle arms [5].  We identified the *pfn-1 (ok808)* as a putative null deletion allele that deletes 1127 bp of *pfn-1*, including 5’UTR and exon 1.  *tiam-1(ntu22)* allele is a nonsense mutation in TIAM-1A(C7STOP) generated by CRISPR/Cas9 and *tiam-1(ntu14)* was introduced two nonsense mutations in TIAM-1A(C7STOP) and TIAM-1B(W7STOP).  The other alleles are putative null alleles, including *mig-2(mu28)* [6], *unc-60(su158)* [7], *wve-1(ok3308)* [8], *fhod-1(tm2363)* [9], *wsp-1(gm324)* [10], *tiam-1(ok772)* [11], *act-4(dz222)* [11], and *unc-119(ed3)* [12]. |

**List of plasmids used in transgenic experiments**

| Plasmid | |
| --- | --- |
|  | *Pser2prom3::tagRFP::tba-1* (kind gift of Hannes E Bülow lab) |
|  | *Pser2prom3::tagRFP::UtrCH* (kind gift of Hannes E Bülow lab) |
| pCL14 | *Pdes-2::TIAM-1 N539* (539 aa)  *TIAM-1 N539* was amplified with *Nhe*I and *Kpn*I sites from cDNA library with the following primers: gaaagggctagcatgggctcacgcctctc and gaaaggggtaccctacgccaactttgcacagcc |
| pCL16 | *Pdes-2::TIAM-1B* (569 aa)  cDNA of *TIAM-1B* was amplified with *Nhe*I and *Kpn*I sites from cDNA library with the following primers: gaaagggctagcatgaattcgtttcgaatatggcg and gaaaggggtaccctattttgaatt tcgatgttccttagagac |
| pCL17 | *Pdes-2::TIAM-1C* (453 aa)  *TIAM-1C* was amplified with *Nhe*I and *Kpn*I sites from *yk1684a10* cDNA clone (kind gift of Prof. Yuji Kohara) with the following primers: gaaagggctagcatggtacaactacgtttcatactacg and gaaaggggtaccctattttgaatt tcgatgttccttagagac |
| pCL40 | *Pdes-2::unc-119::mCherry*  The *unc-119* fragment was amplified from cDNA library with the following primers:  gaaaggggcgcgccatgaaggcagagcaacaacaac and gaaaggggtaccgcatcatacgagtagtcggcc |
| pCL55 | *Pdes-2::unc-119*  The *unc-119* fragment was amplified from cDNA library with the following primers:  gaaaggggcgcgccatgaaggcagagcaacaacaac and  *gaaaggggtaccttatgcatcatacgagtagtcggcc* |
| pCL62 | *Pdes-2::gfp::UtrCH*  The fragment of *UtrCH* was generated from [*Pser2prom3::tagRFP::UtrCH]* with the following primers:  gaaaggggtaccaccatggccaagtatggagaac and gaaaggccgcggttagtctatggtgacttgctgagg |
| pCL63 | *Pdes-2::unc-119::VN173*  The VN173 fragment with linker (GGGGSGGGG) prior to the start codon was cloned into *Pdes-2::unc-119::mCherry* (pCL40) with *Kpn*I and *SacII.* The VN173 fragment was amplified from pAH120 (gift of Prof. Ao-Lin Hsu) with the following primers:  gaaaggggtaccgatgggatccggaggaggaggatccggaggaggaggaatggtgagcaagggcgag and gaaaggccgcggctactcgatgttgtggcggatc |
| pCL64 | *Pdes-2::TIAM-1A::VN155*  The VN155 fragment with linker (GGGGSGGGG) prior to the start codon was cloned into *Pdes-2::TIAM-1A* (889 aa)*::gfp* (pPW12) with *Kpn*I and *SacII.* The VN155 fragment was amplified from pAH121 (gift of Prof. Ao-Lin Hsu) with the following primers: gaaaggggtaccaggaggaggatccggaggaggaatggacaagcagaagaacggcatc and gaaaggccgcggttacttgtacagctcgtccatg |
| pCL67 | *Pdes-2::VC155* (aa 155-239, A206K)  VC155 was amplified from pAH121 (gift of Prof. Ao-Lin Hsu) with the following primers: gaaagggctagcatggacaagcagaagaacggc and gaaaggccgcggttacttgtacagctcgtccatg |
| pCL68 | *Pdes-2::VN173* (aa 1-173)  VN173 was amplified from pAH120 (gift of Prof. Ao-Lin Hsu) with the following primers: gaaagggctagcatggtgagcaagggcgag and gaaaggccgcggctactcgatgttgtggcggatc |
| pHH2 | *Pdes-2::unc-104::gfp*  The *unc-104* fragment was digested with *Kpn*I and *NheI* from *Pitr-1::unc-104::yfp* (pCO131) and cloned into *Pdes-2::gfp.* |
| pHW5 | *Pdes-2::myr::mCherry* |
| pPW12 | *Pdes-2::TIAM-1A* (889 aa)*::gfp*  cDNA of TIAM-1 was amplified with NheI and KpnI sites from cDNA library with the following primers: gaaagggctagcatgggctcacgcctctc and gaaaggggtacctttgaatttcgatgttccttagagac |
| pPW13 | *Pdes-2::TIAM-1A* (889 aa)  cDNA of *TIAM-1A* was amplified with *Nhe*I and *Kpn*I sites from cDNA library with the following primers: gaaagggctagcatgggctcacgcctctc and gaaaggggtaccctattttgaatt tcgatgttccttagagac |

**List of plasmids used in Co-immunoprecipitation experiments**

| Plasmid | |
| --- | --- |
| pYC38 | *pCMV-3xFLAG-UNC-119-MYC*  UNC-119 sequence with *EcoRI* and *XbaI* sites was constructed into p3XFLAG-myc-CMV-26 expression vector (Sigma) to generate pCMV-3xFLAG-UNC-119-MYC (pYC38). The UNC-119 was amplified from *Pdes-2::unc-119* (pCL55) with the following primers: gaaagggaattcaatgaaggcagagcaacaacaacaatc and gaaaggtctagatgcatcatacgagtagtcggcc |
| pYC39 | *pCMV-TIAM-1 N539-HA*  The TIAM-1 N539-HA with kozak sequence (GCCGCCACC) prior to the start codon was amplified with *Kpn*I and *Not*I sites and was constructed into pcDNA3.1(+) vector (Invitrogen) to generate pCMV-TIAM ∆DHPH-HA (pYC39). The TIAM-1 N539-HA was amplified from *Pdes-2::TIAM-1A::gfp* (pPW12) with the following primers: GAA aggggtaccgccgccaccatgggctcacgcctctcatg and aaaggaccggttcaagcgtaatctggaacatcgtatgggtacgccaactttgcacagcc |

| **crRNA sequences** | |
| --- | --- |
| *rol-6* | cgtgtgcgctgccaacaata |
| *tiam-1 a* | ctcatgttcttgctcacaag |
| *tiam-1 b* | caaatgtgtcaaatttgaga |
| **Repair oligo sequences** | |
| *rol-6* | tgtgggttgatatggttaaacttggagcaggaaccgcttccaaccgtgtgcgctgccaacaatatggaggatatggagccactggt  gttcagccaccagcaccaac |
| *tiam-1 a*  (C7stop) | ttctgttgtcagtgagagagaaccgcgttcaaatcagaatgggctcacgcctctcatgttcttgctcacaagccgaatgctggacgaat  gatgaatctacattgaggtt |
| *tiam-1 b*  (W7stop) | aaaagcaaaactcataaaatgctcattaccaaatgtgtcaaatttgagatggcgaaaaccaatggaaacacgtgacatgaacacaatgg  gatcatcaatggc |

**References:**

1. Chen Y-C, Huang H-R, Hsu C-H, Ou C-Y. CRMP/UNC-33 organizes microtubule bundles for KIF5-mediated mitochondrial distribution to axon. PLoS Genet. 2021; 17(2):e1009360.

2. He L, Kooistra R, Das R, Oudejans E, van Leen E, Ziegler J, et al. Cortical anchoring of the microtubule cytoskeleton is essential for neuron polarity. Surrey T, Malhotra V, Baas PW, Bennett V, editors. Elife. 2020; 9:e55111.

3. Yang F-J, Chen C-N, Chang T, Cheng T-W, Chang N-C, Kao C-Y, et al. phiC31 integrase for recombination-mediated single-copy insertion and genome manipulation in *Caenorhabditis elegans*. Genetics. 202; iyab206.

4. Harterink M, Edwards SL, de Haan B, Yau KW, van den Heuvel S, Kapitein LC, et al. Local microtubule organization promotes cargo transport in *C. elegans* dendrites. J Cell Sci. 2018;131(20):jcs223107.

5. Dixon SJ, Roy PJ. Muscle arm development in *Caenorhabditis elegans.* Development. 2005; 132(13):3079–3092.

6. Tannoury H, Rodriguez V, Kovacevic I, Ibourk M, Lee M, Cram EJ. CACN-1/Cactin interacts genetically with MIG-2 GTPase signaling to control distal tip cell migration in *C. elegans*. Dev Biol. 2010;341(1):176–185.

7. Nomura K, Ono K, Ono S. CAS-1, a *C. elegans* cyclase-associated protein, is required for sarcomeric actin assembly in striated muscle. J Cell Sci. 2012;125(Pt 17):4077–4089.

8. Shi R, Kramer DA, Chen B, Shen K. A two-step actin polymerization mechanism drives dendrite branching. Neural Dev. 2021;16(1):3.

9. Refai O, Smit RB, Votra S, Pruyne D, Mains PE. Tissue-Specific Functions of fem-2/PP2c Phosphatase and fhod-1/formin During *Caenorhabditis elegans* Embryonic Morphogenesis. G3 (Bethesda). 2018;8(7):2277–2290.

10. Sheffield M, Loveless T, Hardin J, Pettitt J. *C. elegans* Enabled Exhibits Novel Interactions with N-WASP, Abl, and Cell-Cell Junctions. Curr Biol. 2007;17(20):1791–1796.

11. Tang LT, Diaz-Balzac CA, Rahman M, Ramirez-Suarez NJ, Salzberg Y, Lázaro-Peña MI, et al. TIAM-1/GEF can shape somatosensory dendrites independently of its GEF activity by regulating F-actin localization. Elife. 2019; 8:e38949.

12. Maduro M, Pilgrim D. Identification and cloning of *unc-119*, a gene expressed in the *Caenorhabditis elegans* nervous system. Genetics. 1995;141:977–988.
