# Supplementary material for: TIAM-1 differentially regulates dendritic and axonal microtubule organization in patterning neuronal development through its multiple domains
Source: PLoS Genet. 2022 Oct 12;18(10):e1010454. doi: 10.1371/journal.pgen.1010454 (PMC9612824; doi:10.1371/journal.pgen.1010454)
Supplement: Attachment — Submitted filename: response letter to reviewer.docx [file pgen.1010454.s011.docx]

Dear editor,

We have revised our manuscript entitled “TIAM-1 differentially regulates dendritic and axonal microtubule organization in patterning neuronal development through its multiple domains” to address specific points made by all reviewers. Below is the detailed list of our responses to reviewer’s comments with the description of changes in our manuscript.

*Reviewer #1: In the current Manuscript Lia and coworkers find that the actin regulator TIAM-1 has an interesting effect on PVD neuron development. In the tiam-1 mutant dendrite branching is reduced whereas the axon extends. They show that TIAM-1 interacts with a member of the cortical anchoring complex that connect the microtubule cytoskeleton to the cortex. In this way the regulation of the actin and microtubule cytoskeleton might be connected and may be differently regulated in the axon vs the dendrite. This would be an interesting message, however I think that current manuscript does not connect enough TIAM role in actin (not shown except for branching) to its role on MTs (potentially via UN-119). More importantly, I cannot properly assess the experiments presented in this manuscript. For most of the quantifications presented, I cannot find how this was performed and what the numbers are.*

Detailed methods of quantifications including neuronal morphology analysis (Fig 1, 2, and 7 and S1-S2 Figs), quantifications of mitochondria and synaptic vesicles (S3 Fig), motor fluorescence (Fig 3 and S3Fig), TIAM-1 localization (Fig 4 and S4 Fig), cytoskeletons (Fig 5), UNC-119 and UNC-33 (Fig 6), and EBP-2 comet direction (Fig3) are explained in the section “Quantification and statistical analysis” in Materials and Methods from line 464 to 528. These numerical data are provided in a supplemental file (S2 Table).

*To give a few examples: F2G,L,M I have no idea what and how this was quantified. In L for example how come the motor does not accumulate in the axon in wildtype? Is this really what the authors see?*

Mitochondria and synaptic vesicle numbers were counted as described (line 469-475). Plus-end kinesin motors UNC-104 and UNC-116 generally accumulate at the tip of neuronal processes with plus-end-out MT orientation [24, 58]. In PVD neurons, both axon and the posterior dendrite have plus-end-out MTs (Fig 3D) [59], and therefore, we indeed observed UNC-116::GFP and UNC-104::GFP accumulated at the ends of both axon and the posterior dendrite in wild type (around 36% of UNC-116::GFP signals at axon tips) (Fig 3A and S3J Fig). In contrast, *tiam-1* mutants have very weak signals at posterior dendritic tips with most of plus-end motor signals at axonal tips (Fig 3B and S3K Fig).

*Fig 5C-D How as this quantified? Did the authors trace the whole neuron? But then how did they work around the autofluorescence. Are these maximum projections or sum projections of the neuron Fig 6C,F where and how was the intensity quantified? Is it average intensity? Because I don’t really see it back in the figure.*

In Fig 5, RFP::TBA-1 and GFP::UtrCH were quantified as described from line 495 to 505. We indeed traced the whole neuron by maximum projection images to get total intensities of RFP::TBA-1 and GFP::UtrCH and substracted autofluorescence background of worm body to have signal intensity in each compartment.

In Fig 6, UNC-119::mCherry and UNC-33::GFP were quantified as described from line 506 to 515. For Fig 6C,F, maximum projection images and draw tools were used to define region of intertest (ROI) to measure mCherry and GFP signals in axon and 60 μm of the proximal anterior primary dendrite and then substract background autofluorecence. The UNC-119::mCherry and UNC-33::GFP intensity were divided by the area of ROI to calculate average intensity. The relative ratios of axon to dendrite average intensity of UNC-119::mCherry and UNC-33::GFP were analyzed in Fig 6C and 6F.

*Still here are a few extra thoughts
- The author studied the functions of TIAM-1 domains and conclude that the EVH1 domain is required for development of high order dendrites, and PDZ is responsible for primary dendrites, and the C-terminal region DHPH regulates the axon. Instead of quantifying total dendrites length (Figure2F), quantification of primary dendrite and sidebraches could be separated to better discriminate between actin rich neurite regions vs MT rich neurite region.*

As reviewer#1 suggested, we separated quantification of total dendrites into primary dendrites and high-order dendrites as revised Fig 2F and 2G.

Fig 2

*B isoform seems to be much more important for general neuron development. However the precise contribution (vs A) would need a B specific mutant. Especially considering the very subtle defect of the A isoform. Rescues with the N-terminus is pretty subtle and I would draw too much conclusions based on this.*

It is technically difficult to generate a B form specific mutant since the open reading frame of the B form completely overlaps with the A form. Therefore, it is very hard to disrupt B isoform expression without damaging A isoform. We have tried to target the start codon of the B form, which is quite far from neighboring PAM sites, by different guide RNAs, but all our attempts failed. In order to understand the role of B form, we also generated a single-copy insertion line to stably express B form in *ok772* and found the phenotype is very similar to *ntu22*, and its activity in rescuing high-order branches is not as good as A form (S2D Fig). These results are consistent to our original findings.

*Considering the strong morphology defect, quantifying % axon Mitochodria seems not correct to suggest a transport defect. Maybe this is just a consequence of the shorter dendrite and longer axon.*

We analyzed the density of mitochondria (number of mitochondria/ the length of the neurite) in dendrites and axons (see graphs below). We found that the density of mitochondria significantly decreased in dendrites in *tiam-1(ok772)* mutant. While the number of axonal mitochondria as well as the axon length increased by about twofold in *ok772*, the density of axonal mitochondria remains similar to wild type. It is consistent to the finding that axonal microtubules are significant increased in *ok772* (Fig 5 and Fig 3H) since more plus-end-out axonal microtubules presumably support more kinesin-mediated traffic along with more axon outgrowth.

*“These expression patterns revealed that TIAM-1 localizes at the interface between MT and actin filaments.” and Figure 4: TIAM overlap with MT/actin; in neurites these will always overlap. Therefore not the best to prove connection. Maybe some life imaging would be nice to show co-movement. Alternatively try some actin depolymerization.*

In revision, we carefully examined the localization of TIAM-1::GFP with MT (RFP::TBA-1) and actin filaments (RFP::UtrCH) in the cell body (Fig 4). Z-stack Airyscan confocal images were acquired and analyzed (as described in line 456-461 and line 485-489), which revealed that TIAM::GFP colocalized with MT and actin filaments (with 81% TIAM::GFP intensity colocalized with MT and 57% TIAM::GFP intensity colocalized with actin filaments). In contrast, GFP alone had only 26% colocalized with MT and 18% colocalized with actin filaments.

Fig 4

In our attempts for co-movement by live imaging, we found they were relatively static without much movement. To further examine the relationship between TIAM-1 and actin, we further genetically disrupted the actin structure by using *act-4/Actin* mutant allele *dz222*, which was reported to disrupt dendritic arborization and actin filaments in PVD neurons [46]. In *act-4(dz222)* mutants, while the cortical actin bundles were disrupted, we found decreased TIAM-1::GFP in cell cortex and aggregated TIAM-1::GFP signals with remaining actin structures (S5C Fig). With 3D surface reconstruction of cell surface and TIAM-1 puncta, the distance between TIAM-1 and cell surface was significantly increased in *act-4(dz222)* mutants (S5D Fig). These results suggest that cortical actin bundles are required for TIAM-1 localization in cell cortex under cell membrane.

S5 Fig

*Can unc-119 overexpression suppress tiam-1 phenotypes?*

We overexpressed UNC-119 in *tiam-1(ok772)* mutants and found it cannot suppress *tiam-1* phenotypes, suggesting, without TIAM-1, UNC-119 alone is not enough to support dendritic growth.

*tiam-1(ok772);Ex[Pdes-2::unc-119]*

Scale bar:20μm

*- Where is the wildtype in 2F*

Fig 2F has been revised to better present wildtype data.

*- “Since UNC-119 mediates the link between MTs and actin filaments through UNC-33/CRMP and UNC-44/ankyrin in the cortical anchor complex “ that paper did not show any direct actin link.*

We revised the sentence into “Since UNC-119 is required for microtubule organization [25]” in line 254.

*- “The plus-end-directed Motor Kinesin-1 Prevents plus-end-out MTs from distribution in dendrites in C. elegans” – this is not the latest Shen lab model for kinesin-1*

Line73-74 were revised to add a latest reference [ref 19].

*- Are the mutants use null mutants?*

Except the gain-of-function allele *act-3(st15)*, all other mutant alleles are null alleles (all alleles are described with references in S1 table).

*Reviewer #2: This manuscript by Lin et al investigates the role of the RacGEF TIAM-1 in controlling axon and dendrite development of the multi-dendritic PVD neurons of C. elegans. While others have investigated the role of this protein in axon and dendrite development, the strength of this paper is it’s finding of the opposite effects of a single gene on growth of the axon and dendrite in a single neuron. I found this manuscript to make a valuable contribution to our understanding of mechanisms controlling differential neurite development. I do suggest several changes below that I think will strengthen the manuscript. However, the only concerns that may require additional experimentation are items 1 and 2 below that will involve testing the functionality of the fluorescent reporter already made and an additional examination of isoform specific localization.

1) The authors report the localization of a TIAM-1::GFP reporter driven in the PVD neurons. However, I did not see an examination of the ability of this reporter to rescue any of the tiam-1 mutant phenotypes. Also, it wasn’t clear from the text or the supplemental table whether the cDNA used for this construct was the TIAM-1A isoform.*

We used TIAM-1A isoform as full-length cDNA to generate TIAM-1::GFP reporters (revised as TIAM-1A::GFP). The TIAM-1A::GFP is able to rescue *tiam-1(ok772)* mutant phenotypes quite well.

*tiam-1(ok772);Ex[Pdes-2::TIAM-1A::gfp]*

Scale bar:20μm

*2) A major component of this story is the differential impact of TIAM-1 isoforms on axon vs dendrite development. The authors accomplished this through isoform-specific rescue. I was hoping to see whether the isoforms are differentially localized and if this may contribute to the different actions. Development of isoform-specific reporters would enhance interpretation.*

We have made reporters of all three isoforms: TIAM-1A::GFP, TIAM-1B::GFP, and TIAM-1C::GFP as well as the N-terminal fragment reporter TIAM-1N539::GFP. Different from the dendritic punctate pattern of TIAM-1A::GFP, TIAM-1B::GFP diffusely localized mainly in primary dendrites, the cell body and the axon, while TIAM-1C::GFP mainly localized in the cell body with very low levels in neurites (S4A-C Fig). TIAM-1N539::GFP was very similar to TIAM-1A::GFP with a dendritic specific punctate pattern (S4D Fig). These data suggest that the N-terminal EVH1 domain localizes TIAM-1 to dendrites as punctate pattern, and therefore TIAM-1B mainly acts in primary but not high-order dendrites.

S4 Fig

*3) The manuscript suffers from many grammatical errors that distract from an otherwise informative text. While many of the errors are minor and I could interpret the authors meaning, it created a more challenging read than necessary.*

We apologize for these errors and really appreciate all these efforts and valuable comments from all reviewers and editors.

*4) There were several instances where I disagree with the authors methods for data analysis or noted inconsistency in their analysis of the data.
a. It was unclear what level of ɑ the authors consider as statistically significant. For example, on page 11, the authors indicate that disruption of fhod-1 caused only a slight change in axonal or dendritic growth whereas the unc-60(su158) mutant had significant axonal overgrowth. However, both showed a statistical difference at p<0.05.*

We revised line 125-127 to better describe mutant phenotypes with numbers.

*b. The isoform-specific rescue of axon length suggests a more nuanced interpretation than the authors give. While TIAM-1A and TIAM-1B provided full rescue, TIAM-1C only partially rescued the axon length. This could be due to the inherent variability in transgene expression. Alternatively, it could suggest that the EVH1 and/or the full PDZ domain are required. These alternatives are not recognized in the model of Fig 2H. Similarly, the authors state that the TIAM-1N539 construct restored dendritic development; however, this rescue appears slight compared with the full rescue shown by TIAM-1A and 1B.*

In order to have stable and consistent expression of isoforms, we further generated single-copy insertion strains, which have a single copy of the transgene inserted at the same locus of the chromosome. In agree to the reviewer’s comment, we consistently observed that *TIAM-1C* is not as effective as *TIAM-1A* and *TIAM-1B* in suppressing axonal length, and *TIAM-1N539* is still much weaker to restore dendritic development than *TIAM-1A* in single-copy insertion transgenic strains (S2C-I Fig). Therefore, while the C-terminal fragment (TIAM-1C) and the N-terminal fragment (TIAM-1N539) are able to regulate axonal growth and dendritic development respectively, they still apparently act better together as a whole unit (TIAM-1A). The figure legend of Fig 2I was revised to describe these effects.

S2 Fig

*c. I was appreciative of the authors use of relative dendritic length for many of the measurements—this appropriately recognized that the change in body length may make an absolute measurement of dendritic length misleading. I recommend they also consider relative length for the measurement of the number of synaptic vesicles in the axon (Fig 3E).*

As suggested, we added the analysis for the density of synaptic vesicle clusters (number of synaptic vesicle clusters/axon length) (S3H Fig). We found the density of synaptic vesicle clusters is not significantly different in *ok772* as the case of mitochondria discussed above.

*d. Unlike the use of relative dendritic length, I was not enthusiastic about the authors use of relative fluorescence among cell compartments in Figure 5. This type of analysis could be misleading if the total fluorescence changes in only one compartment. For example, if the fluorescence gets much brighter in the axon, but stays the same in the dendrite this analysis will show a decrease in fluorescence in the dendrite even though there was no real change. Similarly the measurement of* *axon/dendrite ratio of UNC-119::mCherry and UNC-33::GFP does not discern whether the change in expression occurs in one compartment or both. Nor does it appear to account for relative length differences between WT and the mutant.*

The reporter strain of RFP::TBA-1 and GFP::UtrCH carries inherent variability that causes individual difference and inconsistency when individual compartments were directly compared in our statistics. However, when we calibrated signals of each compartment with total signals of all three compartments for individual animals, the signal ratios were very consistent to reveal the effect of *tiam-1* mutant (Fig 5C-D).

For UNC-119::mCherry and UNC-33::GFP, we measured average fluorescence intensity (signals/area of ROI) to exclude the effect of axon/dendrite length differences (line 506-515). The average intensity of axonal UNC-33::GFP was significantly increased in *tiam-1* mutant, while dendritic UNC-33::GFP, axonal UNC-119::mCherry, and dendritic UNC-119::mCherry were not significantly different (see the figure below). However, axon/dendrite ratios of UNC-119::mCherry and UNC-33::GFP are significantly increased in *tiam-1* mutant (Fig 6C,F).

*e. The interpretation of TIAM-1 being associated with actin and attaching to MT seemed a stretch. While the images certainly show localization in the same area, F-actin appeared to be so widely expressed throughout the neurites that a specific association is difficult to determine. The resolution of the Airyscan super-resolution microscope is approximately 150 nm laterally and 400 nm axially. This is much greater than the expected size of F-actin or RacGEF. Therefore, co-localization via light microscopy alone cannot be used to confirm a direct interaction.*

We agree with that the association is difficult to determine in neurites. Therefore, we turn to the cell body to examine the association with actin bundles and MTs. With the resolution of Airyscan, TIAM-1::GFP colocalized with RFP::TBA-1 and RFP::UtrCH significantly better than GFP control (Fig 4B-E). It suggests that TIAM-1 puncta are associated with both MT and actin bundles in some way rather than randomly distributed in cell body.

*5) Other minor issues:
a. The authors should clearly define what is meant by higher-order dendritic branches.*

In this study, we define high-order dendrites as all side branches on primary dendrites (definition added in line 116-119 and Fig 1A).

*b. I assumed that the tiam-1(ok772) is a putative null. If so, this should be stated.*

The *tiam-1(ok772)* is a putative null allele (line 147).

*c. Line 142: The authors should quantify the number of high-order dendrites in the TIAM-1B rescue.*

As suggested, high-order dendrites were quantified as Fig 2G.

*d. The introduction does not put the study in context of other C. elegans studies on TIAM-1. While the authors bring these up in the discussion, it would be valuable to include in the introduction.*

These studies were included in introduction (line 100-103)

*e. Line 161: Provide citation for* *RAB-3 and TOMM-20 labeling* *synapses and mitochondria, respectively. The authors cite Knobel et al for the RAB-3 construct; however, I did not see mention of RAB-3 in this article.*

Cited in line 181 [ref 54 and 55].

*f. Line 169: Provide citation for transport of synaptic vesicles and mitochondria by kinesins.*

Cited in line 190 [ref 24, 56- 57].

*g. Line 173: Provide citation for microtubule orientation in PVDs.*

Cited in line 192 [ref 59].

*h. Figure 4A. The authors state that TIAM-1::GFP was localized in the primary dendrite. Based on the image, it was difficult to determine if the posterior primary dendrite also contained this expression.*

*tiam-1(dz265)*

myr::mCherry

In our observation, TIAM-1::GFP puncta in the posterior primary dendrite are less than the anterior dendrite, but are still present (marked with arrowheads in Fig 4A as well as S4A Fig).

*i. It may be beneficial to test the CRISPR-generated (ntu) mutants in the examination of UNC-119 expression. Is a specific isoform required for expression?*

We found UNC-119 expression is not affected in *ntu22*, while axonal UNC-119 is increased in *ntu14*, very similar to *ok772*.

*j. Line 258: It would be useful to request the Bulow lab CRISPR mutant lacking GEF activity to definitely test if this activity within the C-terminus is required for axon development.*

We have examined the mutant *dz265,* and it showed no detectable phenotype, suggesting the GEF activity is not required for axon development. Therefore, the C-terminal GEF domain controls axon development probably through other mechanism instead of GEF activity.

*tiam-1(dz265)*

*k. Many of the images are missing scale bars. If all images in a single figure use the same scale, this should be stated in the legends.*

All images are revised to have a scale bar.

*l. The KLP-15::GFP signal is difficult to discern due to background autofluorescence.*

In revision, the KLP-15::GFP graph was replaced by EB protein growth direction analysis to directly show microtubule polarity (Fig 3).

*Reviewer #3: The authors characterize the effects of different actin regulators on PVD axon and dendrite development in C. elegans. TIAM-1, a RacGEF, differentially regulates both axon and dendrite growth in vivo. TIAM-1's EVH1 and PDZ domains are required for dendrite development and its C-terminal DHPH domain is required only for axon development. In tiam-1 (ok772) mutants the distribution of actin and microtubules is altered. TIAM-1 is present in the same complex with UNC-119, and genetically interact to control axonal length/outgrowth.

Several concerns should be addressed so that data support the conclusions drawn before acceptance. Authors make claims of Tiam-1's role on the cytoskeleton and polarized cargo distribution. It is important that authors assess microtubule orientation, please exercise care in interpreting localization studies between TIAM-1 and cytoskeletal proteins and changes in organization of cytoskeleton markers. These experiments are the cornerstone on which interpretations of genetic interactions depend. Currently data as presented in these experiments do not support conclusions. More detailed comments are given below.

Major concerns
(1) In line 133, authors mention that the allele ok772 disrupts all the transcripts of tiam-1. Please provide experimental evidence/reference that shows that this allele disrupts all transcripts. This is important for the isoform-specific rescue experiments presented later in the study.*

Demarco et.al have shown that *ok772* has an 838-bp deletion and an 18-bp insertion to remove DH domain of all three transcripts and cause a frame shift [ref 44]. We found that all three transcripts are not present in *ok772* mutant by RT-PCR experiments (data shown below).

*(2) In Fig 1 F, G, H the representative image wve-1(ok3308) shows a much longer total dendritic length as compared to tiam-1 (ok772). However in the accompanying graph in fig 1 H, tiam-1 (ok772) shows total dendritic length similar to wve-1(ok3308). It would be good to show representative images that reflect the data presented in the graph.*

Thanks for the notice, we found a part of *wve-1* dendritic length data left in data processing due to an error in our excel sheets. We have corrected it in the revision (Fig 1H).

*(3) In line 165,166,167 and data presented in Fig 3 A, B and E an increase in the number of SV clusters and changes in mitochondrial numbers could arise from changes in length of the neuronal processes, differences in levels on motors on cargo surface, altered retention etc. Authors present a very narrow interpretation that tiam-1 plays roles in polarized recruitment of cargo. It is unclear how authors exclude other interpretations of the cargo distribution data merely on the strength of the UNC-116::GFP and UNC-104::GFP motor distribution. It is important for authors to examine EB growth direction* *in PVD axons and dendrites in tiam-1 to determine if the polarized distribution of microtubules remains unchanged in tiam-1.*

As suggested, we examined the EB growth direction in PVD axons and dendrites. In wild type, EBP-2 comets grew in retrograde direction in the anterior primary dendrite and grew in anterograde direction in the axon and in the posterior primary dendrite (Fig 3D). In *tiam-1* *(ok772)* mutant, EBP-2 growth direction remained the same as wild type in the anterior primary dendrite and the axon, but most EBP-2 comets grew in a reversed direction (retrograde) in the posterior primary dendrite compared to wild type (Fig3 E-G,I), indicating that microtubule polarity is reversed in the posterior dendrite of *tiam-1* *(ok772)*. Furthermore, we observed that axonal EBP-2 comets significantly increased in *tiam-1* mutant, suggesting that there are more growing microtubules in the axon in tiam-1(ok772) mutant (Fig 3H). Taken together, these results suggest that TIAM-1 is required for regulating polarized microtubule organization of the posterior dendrite and the axon, consistent to changes of cargos and motor protein distribution.

Fig 3

*(4) Greater care needs to be taken in interpreting data presented in Figure 4, currently the data do not support the conclusions drawn from these experiments. The resolution of images presented do not allow one to conclude that Tiam-1 (stated in line 191) is present on the "inner surface of cortical actin bundles (labeled by RFP::UtrCH) (Fig 4B) while they also attached to MT bundles in the cell body". How do authors rule out that the signals ovelap just by chance and do not reflect close juxtaposition. In the case of actin, the actin bundles are present along narrow processes and any signal present in that region including for instance some cargo could well be present in areas that show actin signal. Likewise, in the cell body most Tiam-1 is not present closely associated with microtubules. Lines 192-194, Authors claim that TIAM-1 localizes to the basal region of actin-rich bundles, but in fig 4D, TIAM-1::GFP punctate are present in regions where RFP::UtrCH intensity is low. In the experiment with Ptrn-1, some quantitation where the number of puncta that are present adjacent to each other and the number that are present separately would be very useful to present. There is insufficient evidence from the mere co-localization between UNC-119 and Tiam-1 to support the claim that “TIAM-1 localises at the interface between MT and actin filaments”. The regions of UNC-119 enrichment could also be just changes in diameter of the process or accumulations unrelated to actin-microtubule interfaces. It would be helpful if authors alter the microtubule-actin junctions show an altered Tiam-1 localization.*

We are grateful for these suggestions. In order to estimate the relationship between TIAM-1 and cytoskeletons, we carefully quantified the colocalization of 3D data in the cell body (Fig 4B-E). By comparing TIAM-1::GFP puncta with control GFP protein (freely distributed in the cell), a significantly higher percentage of TIAM-1::GFP is colocalized with either TBA-1 or UtrCH than control GFP (Fig 4C and 4E), suggesting the association of TIAM-1::GFP with MT and actin bundles rather than overlap by chance.

Following reviewer’s suggestion, we genetically disrupted actin filaments by *act-4 (dz222)* mutation to diminish cortical actin bundles and found that TIAM-1 localization is altered away from cell membrane in *act-4 (dz222)* mutant (S5C Fig).

We also estimate the colocalization of UNC-119::mCherry and TIAM-1::GFP by comparing with myr::mCherry. A significant higher percentage of UNC-119::mCherry was colocalized with TIAM::GFP than myr:mCherry (Fig 4F-G).

In revision, we remove the data of PTRN-1 and TIAM-1 in order to focus on the colocalization quantification of TIAM-1 with cytoskeletons and UNC-119.

*(5) In figure 5, authors interpret the data of cytokeletal markers in the dendrite and primary dendrite as evidence for a necessary role for Tiam-1 in forming a boundary between the microtubule and actin cytoskeleton. It is unclear how authors conclude this from the images presented. There are changes in actin and microtubule cytoskeleton in the cell body of Tiam-1. The penetrance of these phenotypes and degree of disruption should be presented as a graph. The changes presented in the primary dendrite of tiam-1 could also arise from developmental changes where the processes are wider just an increase in actin and microtubules that their data do support. These conclusions should be revisited or alternate data to support authors claim should be presented.*

We observed that actin filaments are generally enriched in high-order branches (Fig 5A) and localized at cortex regions just under cell membrane in primary dendrites and the cell body in wild type. In primary dendrites, GFP::UtrCH signals were surrounding to axial RFP::TBA-1 (Fig 5A’). In cell body, GFP::UtrCH signals were basically present at outer rim of the cell (Fig 5A’’ and also in Fig 4D). In *tiam-1* mutant, we scored that almost all worms (>90%) to have GFP::UtrCH signals redistributed to primary dendrites and the organization of actin surrounding axial MTs is disrupted in primary dendrites (Fig 5B’ compared to Fig 5A’). About 60% of worms had severely disorganized cytoskeletons in the cell body (Fig 5B’’). In our observation, the *ok772* did not just have increased actin filaments but the disruption of the organization of the peripheral actin domain relative to central MT domain. We do agree that “forming a boundary” seems claim too much for its direct physical role. Therefore, we revised the conclusion to “Taken together, these results suggest that TIAM-1 is required for the proper distribution of actin filaments and the organization of cytoskeletons in PVD neuron.” (line 250-251). We also revised the title for the same reason.

As the reviewer suggested, the penetrance of phenotypes and degrees of disruptions were presented as graphs (S6 Fig).

S6 Fig

*(6) Authors claim that in tiam-1 mutant, UNC-119 distribution increases in the axon. Could this increase merely be a reflection of the increased axonal length? In Fig 6 A and B, tiam-1(ok772) mutant animals show UNC-119::mCherry localization in higher-order dendrites that is absent from wild type images. How does one account for this localisation?*

In revision, the average intensity of UNC-119::mCherry and UNC-33::GFP was quantified to exclude the effect of the length differences between wild type and *ok772*, and then the relative ratios between axon and dendrite were calculated as described in line 506-515 (Fig 6C,F).

In our observation, UNC-119 distributes mainly in primary dendrites and the axon with low levels in high-order branches. Due to individual variability, these signals of UNC-119::mCherry in high-order branches are not consistently higher in *ok772* than wild type.

*(7) In Figure 6L authors see a doublet with anti-FLAG. Are there additional bands? Are these different isoforms of UNC-119? It would be useful if authors provide some explanation for this.*

We consider the lower additional band as partially degraded form of UNC-119, since we expressed the UNC-119 by a cDNA construct, there should be no other isoforms of UNC-119 present. We have revised the Figure legend of 6L for it.

*Minor concerns
(1) The paper identifies a genetic pathway for axon outgrowth, however there is nothing in the introduction about axon outgrowth and the roles of actin and microtubules in this process. This sets up the paper well for the differential roles of TIAM-1 that they describe. Such a paragraph would be very useful especially for a new student/post-doc in the field. I strongly suggest adding that.*

As suggested, we added a paragraph in introduction for axon outgrowth (line 53-60).

*(2) In all figure with graphs with error bars eg Figure 1 H and I do please list the n’s in the legend. It is more useful to present the data as violin plots so that the variability can be assessed.*

We have revised graphs as scatter plot with bar to display all data points and listed sample size in legends.

*(3) Please state how the act-3 gof allele is thought to act. Does it prevent attachment of other actin remodeling complexes or affect the kinetics?*

The *st15* allele was characterized as a gain-of-function mutation that caused actin enrichment in muscle arm termini in the actin gene cluster *act-1,2,3* [Dixon SJ and Roy PJ, 2005 Development, reference 79]. We sequenced the actin gene cluster of the allele and identified a missense mutation Y144N at the *act-3* gene, but the mechanism of how *st15* acts remains unclear.

*(4) Line 142-143, Authors say that “TIAM-1B expression did not rescue distal high-order dendrite morphogenesis defects”. Graphs in Fig 2F suggest that overexpression of TIAM-1B in tiam-1(ok772) partially rescues the total dendritic length. It would be useful to explain this in their interpretation and also consider showing rescue in primary and higher order dendritic length in a supplementary figure.*

As suggested, we have revised the graph into Fig 2F and 2G to show rescue in primary and high-order branches.

*(5) In Fig 6 D and E, several images of differing background intensities are stitched together to reconstruct the PVD neuron. It is unclear if such images were directly used to calculate the protein level ratios presented in the graphs. It would be useful to provide a more detailed description in the methods of their image processing pipeline leading upto the quantitation they present.*

In order to cover the PVD neuron, several image serials were taken and stitched together for further analysis and quantification. We measured signals of axons and dendrites by subtracting background signals of the same image to exclude the effect of background difference among images. The details of image acquisition and quantification were described in methods (line 456-461 and 506-515)

*(6) Authors have used 'cargoes' three times (lines 50, 300, 309) and 'cargos' four times (lines 58, 169, 181, 327). Please use consistent spelling throughout the manuscript.*

All “cargoes” were revised as “cargos”.

We really appreciate all these constructive suggestions and insightful comments from all reviewers to improve our manuscript and thank you for all the effort in the editorial process.
