# Supplementary material for: TIAM-1 differentially regulates dendritic and axonal microtubule organization in patterning neuronal development through its multiple domains
Source: PLoS Genet. 2022 Oct 12;18(10):e1010454. doi: 10.1371/journal.pgen.1010454 (PMC9612824; doi:10.1371/journal.pgen.1010454)
Supplement: Attachment — Submitted filename: response letter to editor.docx [file pgen.1010454.s012.docx]

Dear editor,

We really appreciate all reviewers’ constructive and insightful comments and have revised our manuscript entitled "TIAM-1 differentially regulates dendritic and axonal microtubule organization in patterning neuronal development through its multiple domains". The following is a detailed list of our responses to reviewers' comments with the description of changes in our manuscript.

*Reviewer #1: Overall I’m pretty happy with the extended methods and the extra data/answers to my other point. Also I think the EB analysis and focus on MTs strengthens the paper well.*

*I now mainly have minor points left that should easily be addressed.
Fig S5 - I do not think that an UtrCH is a good marker to mark the cell cortex to define the distance of TIAM to the membrane. Especially in WT the actin filaments may not be at the cortex and thus the actual distance of TIAM to the membrane might be different. In actin depletion, I guess there is more cytosolic marker working as a fill and thus would work better. Therefore I’m not so sure about this statement line 234 “These results suggest that cortical actin bundles are required for TIAM-1 localization in cell cortex under cell membrane.”*

To better focus on the result, we have revised the sentence as “These results suggest that TIAM-1 localization requires appropriate actin organization.” (line 234).

*Fig 4 - Colocalization quantification – Is the whole CB used? If so for WT the main colocalization quantified would be the big blobs on left of the CB which for sure are not MTs. So this does not work.*

In Fig 4, we did perform the quantification with the whole cell body. By manually excluding these big blob regions, we found that TIAM-1A::GFP is still colocalized much better with RFP::TBA-1 in comparing with GFP control as the original quantification (Figure below). Since these results are consistent, we prefer to use the whole cell body to avoid manual bias.

Fig 4C

*Small changes
- UNC-119 comes out of the blue (line 235) – some introduction would be nice or move it to a later paragraph*

A brief Introduction has been added (line 235-236).

*Line 297: “Based on our findings, we propose a working model that TIAM-1 organizes actin filaments and MTs to direct axonal/dendritic development by regulating UNC-119 (Fig 7H).” does this suggest that UNC-119 regulates actin? Based on what data?*

To avoid misunderstanding, we separated the statement into two sentences as “Based on our findings, we propose a working model that TIAM-1 regulates MT and actin organization to direct axonal and dendritic development (Fig 7H). UNC-119 is essential for TIAM-1 to regulate axonal outgrowth.” (line 296-298).

*- Figure 3D-E use of red/blue colors confusing. Blue is defective? Maybe turn colors around?*

As suggested, we revised the color presentation in Figure 3D and 3E.

Fig 3

*In general the manuscript would benefit from having a native speaker correcting the grammar. Here are a few textual suggestions to start with:
- Neurons do not really migrate (line 52); remove ”the” (line 118); remove “had” (line 126); “well MT organization” is not very nice (line210) maybe replace Well by Proper; Line 213 “We found that TIAM-1A::GFP specifically localized TO THE somatodendritic compartment with punctate patterns in THE primary and high-order dendrites but DOES not LOCALIZE TO the axon (Fig 4A).”; remove “Fig” line 244; replace “manifested” line 286 by e.g. elucidated; line 335“Although THE MT”*

We revised the line 52, 118, 126, 210, 213, 244, 286, and 335 as suggested.

*Reviewer #2: The authors have satisfied my concerns in this revision.*

*Reviewer #3: The authors have resubmitted an excellent manuscript that has addressed all my concerns. I do think that the airy scan images are a good addition but still do not necessarily support the localization of TIAM-1 as being associated with actin and microtubule cytoskeleton. Using soluble GFP as a control may not be the best method. Could another microtubule or actin related protein have been used? I think no additional experiments are necessary however, it would be useful to be more careful in what is claimed for these data. An excellent paper and a valuable addition to the literature.*

As suggested, we revised line 224-225 “suggesting that rather than exhibit random distribution, TIAM-1A::GFP is localized to MT and actin bundles” to “suggesting that rather than exhibit random distribution, TIAM-1A::GFP has a better colocalization with MT and actin filaments” and also revised line 237-238 “According to these observations, TIAM-1 is associated with UNC-119, actin filaments, and MTs.” to “According to these observations, it is likely that TIAM-1 is associated with UNC-119.”.
